# A Scoping Review for Usage of Telerehabilitation among Older Adults with Mild Cognitive Impairment or Cognitive Frailty

**DOI:** 10.3390/ijerph19074000

**Published:** 2022-03-28

**Authors:** Nurul Hidayah Md Fadzil, Suzana Shahar, Roslee Rajikan, Devinder Kaur Ajit Singh, Arimi Fitri Mat Ludin, Ponnusamy Subramaniam, Norhayati Ibrahim, Divya Vanoh, Nazlena Mohamad Ali

**Affiliations:** 1 Centre for Healthy Ageing and Wellness (H-Care), Faculty of Health Sciences, Universiti Kebangsaan Malaysia, Kuala Lumpur 50300, Malaysia; hidayahfadzil143@gmail.com (N.H.M.F.); roslee@ukm.edu.my (R.R.); devinder@ukm.edu.my (D.K.A.S.); arimifitri@ukm.edu.my (A.F.M.L.); ponnusaami@ukm.edu.my (P.S.); yatieibra@ukm.edu.my (N.I.); 2 Programme of Nutrition and Dietetics, Health Campus, School of Health Science, Universiti Sains Malaysia, Kubang Kerian 16150, Malaysia; divyavanoh@usm.my; 3 Institute of IR 4.0 (IIR4.0), Universiti Kebangsaan Malaysia, Bangi 43600, Malaysia; nazlena.ali@ukm.edu.my

**Keywords:** telerehabilitation, telehealth, telemedicine, older adults, elderly, cognitive impairment, mid cognitive impairment, cognitive frailty

## Abstract

Older adults are vulnerable towards cognitive frailty that can lead to adverse health outcomes and telerehabilitation appears to be a potential platform to reverse cognitive frailty among older adults. The aim of this coping review is to identify the usage of telerehabilitation and its common platform of delivery among older adults with mild cognitive impairment (MCI) or cognitive frailty (CF). Articles published from January 2015 until October 2020 were selected. Out of the 1738 articles retrieved, six studies were identified. Two articles were randomized controlled trials, one was a pilot study and three were qualitative studies. The outcome suggests that telerehabilitation may improve the quality of life among participants as well as it can be a useful and supportive digital platform for health care. Some types of technologies commonly used were smartphones or telephones with internet, television-based assistive integrated technology, mobile application and videoconference. Telerehabilitation utilization in managing cognitive frailty among older adults is still limited and more research is required to evaluate its feasibility and acceptability. Although telerehabilitation appears to be implemented among older adults with MCI and CF, some social support is still required to improve the adherence and effectiveness of telerehabilitation. Future research should focus on the evaluation of acceptance and participants’ existing knowledge towards telerehabilitation to achieve its target.

## 1. Introduction

Older adults are mostly defined by the age of 60 and above [1]. With advanced global development of information technology, there will be an advantage to apply these technology in improving or extending services to the older population [2]. In the past few years, many senior citizens opted to live with their family members and gradually settled down; however, others may have to live more independently due to several factors such as migration of children post-marriage or children pursuing studies or working in other places [3,4]. Problems arise when they face multiple chronic conditions or acute illnesses, which can increase the likelihood of being dependent on others and affect the quality of life of both the older adults and their caregivers. Integrated health care monitoring technology such as mobile health (mHealth) can overcome these obstacles among the ageing population and promote their wellbeing [5,6].

Telerehabilitation is an alternative platform of telecommunication-based practice that could provide alternatives in delivering health education and care for clients or individuals either in clinical, community or home care settings [7,8]. It is a rapidly developing discipline that has become a key part of telemedicine and e-health [9]. It covers the scope of therapeutic intervention, management of disease, coordination of care, caregiver training and education, patient networking and consultation by multidisciplinary professionals [8,10]. In conformity with research findings, the implementation of telemedicine was feasible in delivering care among older adults [11]. It can enhance the communication between users, enable access to information, accelerate task completion and has an interactive interface [12]. Some challenges in delivering telerehabilitation among older adults were internet access, familiarity towards technology and digital literacy [13].

Cognitive frailty (CF) is considered as a precursor of neurodegenerative processes that involved simultaneous in the presence of frailty in both physical and cognitive domains [14]. It may be prescribed as the occurrence of both cognitive impairment and pre-frailty, before progressing to dementia [15,16,17]. It can also be described by lower grip strength, lower gait speed, weak lower limb muscle strength and impaired delayed recall [15]. The prevalence of frailty among community-dwelling older adults is increasing over the world where by, estimated range from 1.0 to 22.0% [18,19]. Cognitive frailty renders individuals to become more susceptible to adverse health outcomes for instances, falls, other physiological disability, hospitalizations and mortality [19,20,21]. In Malaysia, the prevalence of cognitively pre-frail and frail of multi-ethnic older adults was 37.4% and 2.2%, respectively [22]. There are a number of risk factors related to the occurrence of cognitive frailty including vascular disease, lifestyle factors such as sleep pattern, physical activity, smoking status, psychosocial performance, poor nutritional status, and recently oxidative stress [23,24,25]. Recent findings indicated that malnutrition and depression were related to cognitive frailty [26]. Compared to this study, the other risk factors of cognitive frailty were advanced age, low intake of niacin, limited social support, depression and lower functional status [23].

On the other hand, mild cognitive impairment (MCI) is known as a transitional state between normal aging and dementia [27,28]. As mentioned by Petersen et al. (1999), early detection of MCI can be manifested by memory disturbance and memory disorder among older adults [29]. Recently, a meta-analysis study indicated that the prevalence of MCI among older population was between 3% and 42% globally [30]. In Malaysia, seven out of ten older adults was presented with MCI and more prevalent among advanced age group, low education level, stay alone and low level of life satisfaction [31]. Lower intake of fruits and vegetables and less participation in calorie restriction were also the risk factors of MCI among older adults in Malaysia [32]. In addition, MCI can lead to severe memory regression, physical dysfunction, impaired mental health and low quality of life [33]. There are some interventions to prevent MCI had been introduced such as pharmacological intervention, over-the-counter (OTC) supplementation, physical, cognitive activity and cognitive stimulation and reminiscence therapy [34,35,36,37,38,39]. In later life, enhanced cognitive engagement is related to decreased risk for MCI [40].

The reversal of CF is possible using a multi-domain intervention [23]. Meanwhile, MCI reversion rate to cognitively normal was different among age groups [41]. In the present, there is no reliable evidence that a single intervention can curb this problem as the risk factors are multidomain consisting of physical, nutritional, cognitive and psychosocial aspects [42,43,44,45,46,47]. Improvement of muscle strength and energy are the main indicators of reversal of cognitive frailty when a combined intervention is applied. Presently, a multi-domain intervention is in progress to examine the possibility of reversing cognitive frailty among Malaysian older adults [48].

Similarly, implementation of telerehabilitation among older adults with CF and MCI is still ongoing and had indicated some advantages such as feeling of assurance and safety, providing diagnosis, treatment, education and rehabilitation including access to care either in rural or urban areas, as well as reduce cost for both healthcare providers and patients [49,50]. Nevertheless, there are some barriers that require attention in implementing telerehabilitation among older adults which include self-efficacy, digital literacy, experience, frequency of usage and reliance on guidance [51,52]. Moreover, more randomized controlled trials, larger numbers of participants and trial designs that reduce bias are needed to evaluate effectiveness of mobile health intervention for individuals with cognitive impairment [53]. Study indicated that social support can facilitate older adults to use digital technology [54]. Research implementing telerehabilitation to address CF and MCI among older adults is still limited. There are increasing reports suggesting the need of telerehabilitation services, the development of telerehabilitation interventions and support for people with disabling conditions that potentially limit access to rehabilitation services. In addition, the current COVID-19 pandemic, in which older adults are the vulnerable group, has placed pressure for more services related to better care and communication with older adults to be delivered effectively through telerehabilitation [55]. Thus, in this paper, we aim to conduct a scoping review of studies concerning usage of telerehabilitation among older adults with CF and MCI and their understanding and perception of telerehabilitation.

## 2. Methods

This scoping review was conducted on telerehabilitation implemented among older adults with CF and MCI following the Arksey and O’Malley (2005) framework and the Preferred Reporting Items for Systematic Reviews and Meta-Analyses Extension for Scoping Review (PRISMA-ScR) guidelines [56,57]. The checklist of PRISMA-ScR is attached in this review. The five stages that were applied as described below.

### 2.1. Identifying the Research Questions

The objective of this scoping review is to map existing telerehabilitation among older adults with CF and MCI. The research question was identified to direct the review and determine the existing relevant studies. Therefore, the present scoping review were sought to answer the following research questions:

1. What are the types of technology used and its features among older adults with CF and MCI?
2. What are the outcome measures reported following telerehabilitation in application among older adults with CF and MCI?

### 2.2. Identifying Relevant Studies

#### 2.2.1. Search Terms

We identified relevant studies published between 2015 till 2020 for this scoping review. The PCC (Population, Concept and Context) in Table 1 below was used to guide at this stage based on the following mnemonic PCC, where the target population of this scoping review was older adults with cognitive frailty (CF) initially. However, as this concept was newly defined in 2013 thus related research was still limited [14]. Therefore, in line with its definition of the presence of MCI, the keywords were broadened to related findings suited with MCI instead of CF alone. Similarly, telerehabiltiation keywords were also expanded to include telemedicine, telehealth or mobile health (mHealth). The Medical Subject Headings (MeSH) for key terms related to telerehabilitation and older adults were analyzed carefully by reviewers. The final keywords used in this review were as followed: [“Telerehabilitation” OR “Telemedicine” OR “Telehealth” OR “mobile Health”] for telerehabilitation, [“Cognitive frailty” OR “Mild cognitive impairment”] for cognitive frailty and [“Older adults” OR “Elderly” OR “Aging Population”] for older adults.

The inclusion criteria were full-text articles, written in English, found in peer-reviewed journals, prospective studies that involved understanding, perception or intervention of rehabilitation through internet, videoconference, into the home of persons with mild cognitive impairment or cognitive frailty among older adults aged 55 years old and above. Studies were excluded if they did not fit into one of these categories. In addition, any review articles and all other secondary sources were excluded from the study to make sure that the analysis was restricted to primary data.

#### 2.2.2. Databases

Five databases were queried in this review: PubMed (MEDLINE), Embase (Science Direct), IEEE Xplore and Scopus. We believe these five search databases would be sufficient to gather the relevant journals within the area of interest. A hand search in JMIR Publication was also included because more research related to telerehabilitation interventions were reported in this journal. In summary, 1736 articles were retrieved using the above keywords and databases.

### 2.3. Study Selection

With guidelines from Preferred Reporting Items for Systematic Reviews and Meta-Analyses Extension for Scoping Review (PRISMA-ScR), any duplicated titles were removed (*n* = 30) leading to 1726 articles to be considered. After these articles were reviewed, a total of 1720 of them were excluded due to the wrong study populations which were not involving older adults but only the caregivers or medical providers, and the wrong concept where the interventions were focusing on medication and dietary supplementation. Only six articles were finally included in the review. Methodological quality of individual studies were not conducted as this is not a compulsory in scoping review [58].

### 2.4. Charting the Data

At this stage, all selected data that was extracted from the journal databases and was organized using Microsoft Excel. The extracted data are author(s), publication year, country study design, duration of study, intervention, type of digital technology delivered, features, outcomes and findings. We presented the data in two separate tables under the result section of this review.

### 2.5. Collating, Summarizing and Reporting Results

**Figure A1:**
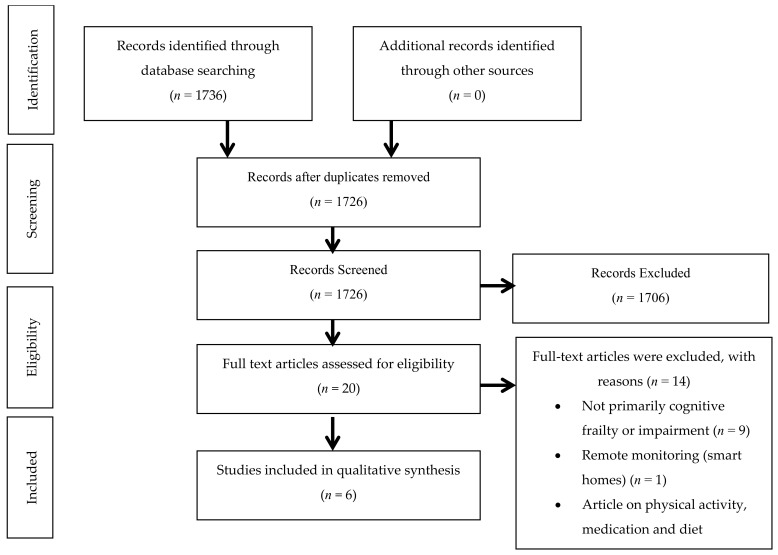
Preferred Reporting Items for Systematic Reviews and Meta-Analyses Extension for Scoping Review (PRISMA-ScR) flow diagram for telerehabilitation among older adults with mild cognitive impairment and cognitive frailty.

In accordance with the Arksey and O’Malley (2005) framework, the last stage was to categorize the relevant findings based on the research questions and focused on measure outcomes of telerehabilitation among older adults with CF and MCI. The flow diagram for this scoping review has been summarized in Figure A1.

## 3. Results

### 3.1. Characteristics of the Selected Studies

Following the framework and the inclusion criteria outlined above, the literature search found six relevant studies (Table 2). All studies were conducted between the year 2018 and 2020. Five of them were conducted among older adults with MCI. These studies were from the United States, Canada, Spain and Sweden. Only one study focused on CF which conducted in Hong Kong. The studies were comprised of three intervention studies (two RCTs and one single-arm study) [42,59,60] and three qualitative studies [61,62,63]. Only one in this selected studies involved older adults and caregivers [60]. The sample size of participants involved ranged between six to 100 participants. The age of participants was between 55 to 78 years. All studies had recruited the participants from community-dwelling settings.

Telerehabilitation was conducted to deliver cognitive training for individuals diagnosed with cognitive impairment [60]. In this cognitive training, participants were required to choose their own goals (at least two or more goals) at the beginning of in-person assessment. Each goal was targeted at every three weeks up to eight weeks. Both in person versus telehealth via videoconference were observed repeatedly during the baseline and treatment phase. On the other hand, mHealth is used to intervene the cognitive function and physical activity among these target population while control group only received conventional method [42]. Individual semi-structured interviews were conducted among qualitative studies [61,62,64]. In the latest studies, the recruitment of participants was accomplished through telephone interview [62]. The session, which was also audio-recorded, took about 15 to 120 min and presented to be feasible among these target population. In these three studies, the objective was to explore the understanding, perception and impact of telehealth or mHealth towards older adults with MCI [61,62,65]. However, assistance from caregivers or guardians were sought to assist answering some of the questions due to their poor memory recall and concentration [42].

### 3.2. Type of Digital Technology, the Features and Outcome Measures of the Selected Studies

This review identified four different technologies used in these studies. They were web-based for educational material, smartphone, video-conference and television-based assistive integrated service [42,59,60,65]. Seven components were incorporated to formulate mHealth intervention among CF older adults such as target behaviors to be altered, personalized goals, motivation, e-reminders, use of valid devices, integration of self-tracking and e-coaching [42,66]. The health coach were required to engage with telephone, email and text messaging to interact and ensure adherence of participants as well as web-based material to assist during learning session [59].

Two intervention studies were focused on multi-domain outcomes [42,59] while one intervention studies highlighted single domain [60]. Studies that were focused on multi-domain features consisted of cognitive, physical activity, nutrition and psychosocial aspects [42,59]. In the Virtual Cognitive (VC) Health Program, participants were required to attend 12-month individually tailored intervention that divided into two phases, lifestyle change and reinforment phase [59]. Each subjects was assigned with a health coach to undergo training that comprised of personalized management of healthy diet, physical exercise, cognitive training and social engagement [59]. Amongst these studies, two of them were conducted from ongoing research in the TV-AssistDem and SMART4MD project, respectively, that focused on older population with mild cognitive impairment or mild dementia [62,65].

The pilot study was aimed to evaluate problems relating to feasibility of the intervention delivered using telerehabilitation on parameters such as recruitment, retention, participation and compliance as well as to distinguish the preliminary effects [42]. The authors reported that there were no previous studies reporting the outcome of eHealth intervention on physical inactivity of cognitive frail aging population. Thus, in this first case, the participants were required to participate in both conventional behavior change intervention and mHealth (smartphone-assisted program using Samsung Health and Whatsapp) intervention. The control group used a conventional behavior change process where they received brief activity counselling, telephone follow-up, health education and exercise training via face-to-face. Smartphones were utilized because of the accurate sensors and validity-tested device to measure step counts. Additionally, the Samsung Health app can coach users to set a goal, review the performance and give reminders [42]. This study was found to be feasible and effective at enhancing compliance with a brisk walking training program.

Most outcomes of these studies included quality of life or functional health patterns, cognitive function, behavioral status, adherence towards medication, eating habits, perceptions of mobile health, use of technology and physical activity. The research outlined participants’ technology literacy, assistance for living independently and maintenance of social contacts which were the three elements considered in designation of mHealth technologies [62]. Many older adults with cognitive frailty regarded themselves as having inadequate technical skills, experience using mHealth and being concern about their data privacy and security [59,62,67]. Older adults who have problems in exploring mobile technology may perceive it as time- and energy consuming to some extent, and required further assistance with device management and technology [42,62]. Other concerns were also raised among older adults which were cost of technology of both the online platform and the device [62]. Older adults were more familiar in contacting health care staff through telephone for booking appointments or being notified for medical records or results as compared to eHealth online system [61].

Telehealth and mHealth were reported to improve the quality of life of older adult with mild cognitive impairment [42,62]. This study also agreed that the mHealth may complement their daily life but somehow they could do without it [62]. In addition, older adults also described mobile technology as: an enabling tool to faster communicate with others; providing a sense of security and helped to stay connected with others; possessing a multipurpose function, including its utilization with reminders, writing notes, taking notes or playing games related to cognitive stimulations [62]. Findings also suggested that mobile health is a distinguished tool to enhance physical activity as evidenced by significant increment in the intervention group as compared to control group in terms of walking time, step count, time of brisk walk and peak cadence [41]. We summarized this section in Table 3.

### 3.3. Additional Information

One study had reported the factors that discourage participation from older adults during recruitment phase [60]. They were inadequate time of caregivers and individuals and negative emotions during assessment and intervention [60]. The researchers found that it was difficult to recruit participants with MCI and were surprised that some chose to engage without a support person [60]. They initially planned to recruit participants from clinical settings but since this approach resulted in low participation, the authors expanded the criteria and recruited from community-based and hospital-based geriatric assessment programs. The assessments were held in one or two sessions according to participants’ availability. Some examples of set goals were to track dates, plans and activities, remember important events, names, storyline from books and reduce frustration related to memory and organizational difficulties.

## 4. Discussion

The purpose of this scoping review is to map the existing evidence on the usage of telerehabilitation among older adults with mild cognitive impairment or cognitive frailty. Our review demonstrated that the mainly used technologies for telerehabilitation were websites, smartphones, video-conference and television-based assistive integrated services as an ICT platform. Older adults perceived digital health care technology as a supportive platform for them to reside independently, have better communication and provide sense of security, but some may require further support to explore the technology. The intervention study using digital health technology from this review indicated that the quality of life among participants improved compared to the control group [42]. In this study, participants in the control group who received conventional behavioral change via face-to-face were reported giving burden to the caregivers [42]. Another study revealed that ICT platforms used to conduct telerehabilitation was suitable to be implemented among MCI patients as drop-out rate in the control group was higher when compared to telerehabilitation intervention group [68].

Furthermore, prior to implementation of telerehabilitation among these target population, we should understand their motivation and readiness towards digital or technology literacy as their generation was raised with different types of technology such as television, radio and telephone compared to current technology such as personal computers, notebook, and tablets including video conferencing and apps. Adults with cognitive frailty are normally older, have lower education levels and different opinions towards information and communication technology (ICT) used [69]. A study was aimed to investigate the readiness towards telehealth among older adults and found that self-efficacy and digital literacy are essential elements to be considered in ICT applications [69]. Lack of technical resources and participants’ condition are also other elements that need to be emphasized for implementation of telerehabilitation despite current modern technology [70]. However, in terms of online interventions, participants’ compliance and adherence towards intervention might be challenging as it is dependent on the social support from family, peers and community [71,72]. Besides that, availability of device is one of the crucial components to conduct telemedicine which can be either through mobile phone, tablet or personal computer [73].

Despite a lack of familiarity with tablet computers, older adults were comfortable with the technology [74]. It was reported in a systematic review that most interventions related to mobile health applications highlighted improvement in their evaluation on health outcomes among people with Mild Cognitive Impairment (MCI), Alzheimer’s disease and dementias [53]. This is in line with a research reported that telemedicine is an applicable tool to nurture older adults efficiently in reducing risk of depressive symptoms and enhancing social functioning and also improving cognitive and nutritional statuses [75]. This contributes to reducing vascular diseases and exacerbation of pre-existing chronic illnesses. In respect to improving cognitive status via the use of technology, more recent research has reported that the main methods and technologies used were telemedicine, virtual reality, augmented reality and digital games [76].

People with cognitive impairment who utilize digital technology may have better health outcomes and statuses with less depression and sociodemographic variables [64]. A study revealed that older adults utilize digital technology to support their memory [64]. They were enthusiastic to utilize digital technologies if they could recognize their benefits and had access to the technology [77]. Older adults who receive education via ICT platform enhanced their nutrition and cognition function [59,78]. In general, this population utilizes smartphones and tablets more frequently, but has limited capability to use specific apps or software to support memory [73]. Mobile technologies could impart the basic physiology and security but lack in self-regard and agency [79]. Most studies reported smartphone technology with text messaging was among the most preferred and available to be used in ICT based intervention or application to improve health [80,81,82,83]. Healthcare providers sending notifications through SMS or short message service was accepted and suitable to be utilized [84]. Nevertheless, a senior friendly web-based application targeting community dwelling senior citizens was also reported as a feasible tool to educate older adults about non-pharmacological approaches for memory improvement [85]. Further explanation related to benefits of using digital technology in delivering online education must be conducted among these target population. To ensure their compliance, assistance during session must be provided as well as send reminders and notifications [86]. Participants were also required to answer adherence questionnaire to determine their understanding towards related topics [86].

Virtual Cognitive (VC) Health Study has indicated that a digital intervention can be carried out from baseline until completion with presence of accessibility of internet-connected device [44]. Some of the participants who did not own any devices could still access information and internet technology nearby in libraries or community centers [44]. In addition, the researchers also affirmed that adoption of digitally delivered multi-domain lifestyle interventions provides an opportunity to lessen people’s medical burden and delay the onset of disease, but whether it may alleviate the prevalence of disease remains unknown [44]. Most multi-domain trials were conducted in a face to face mode such as Finnish Geriatric Intervention Study to Prevent Cognitive Impairment and Disability (FINGER), Multidomain Alzheimer Preventive Trial (MAPT), Prevention of Dementia by Intensive Vascular Care (preDIVA) and Sub-study of Systolic Blood Pressure Intervention Trial (SPRINT-MIND) [24,87,88]. One of the requirements in both FINGER and PreDIVA was commitment to a clinic appointment which could have restricted involvement of older adults who had difficulty to attend because travel costs and physical limitations to their mobility. Other present ongoing multidomain trials that are connected to WorldWide-FINGERS initiative are examining Internet-based interventions [89]. For example, Maintain Your Brain (MYB) which targeted multiple risk factors for dementia and Alzheimer disease using web-based technology among Australian older adults over 3 years [90]. In this trial, the intervention domains emphasized on physical activity, nutrition, cognition, mental health like depression and lifestyle risk factors such as smoking and heavy drinking. Participants in the intervention group received the module consisting of Dubbed “Physical Activity”, “Nutrition’, “Peace of Mind” and “Brain Training”. In addition, other protocol randomized controlled studies with multi-domain of telerehabilitation intervention were also outlined to conduct the same goal which is to counteract cognitive dysfunction and enhance quality of life such as Games for Older Adults Active Life (GOAL) Project and TV-AssistDem [65,91]. Telerehabilitation was found to be feasible among people with mild cognitive impairment or vascular cognitive impairment [68].

Some limitations needed to be addressed in this review. Firstly, the authors identified the low number of articles being analyzed in this scoping review. The term of cognitive frailty was newly introduced in 2013 and the articles related to this topic is still limited. Secondly, since the target population was cognitively frail older adults, interaction with their caregivers’ have not been considered in most of the studies. Previous studies reported an encouraging future among caregivers in managing dementia patients using in-home telehealth. Hence, the authors suggested that future studies should address the implications of telerehabilitation intervention among cognitively frail patients, their caregivers, healthcare professionals and stakeholders.

## 5. Conclusions

This review found the existing technology used in telerehabilitation are telephones or smartphones with internet features, followed by television-based assistive integrated technologies, mobile applications and video conferencing. Telerehabilitation among older adults with CF and MCI may be possible to be an encouraging platform for older adults to live independently, provide social interaction and a sense of security. Intervention studies that utilized digital health technology demonstrated that quality of life among this population could be enhanced. However, the available evidence is still lacking and more studies are required to evaluate its feasibility and acceptability, particularly using larger samples and involving those from a low socioeconomic status. An evaluation towards readiness and barriers of using telerehabilitation and informative communication technology platforms should also be conducted prior to the development of such intervention. Future research should focus on the evaluation of acceptance and participants’ existing knowledge towards telerehabilitation to achieve its target as well as determine which interventions have an absolute effect on certain outcomes.

## Figures and Tables

**Table 1:** Table of PCC.

Population	Older adults
Concept	Telerehabilitation
Context	Older adults with cognitive frailty (CF) or mild cognitive impairment (MCI)

**Table 2:** Characteristics of the selected studies.

Author/Year/Country	Study Design/Duration	Subjects	Intervention
Bott et al. (2018) United States	Single arm, pre-post pilot study 12 months	82 participants with cognitive impairment. Mean age was 64.0 year.	Intervention group: Virtual Cognitive Health program
Burton & O’Connell (2018) Canada	Randomised Control Trial 2 months	6 subjects with cognitive impairment. Mean age was 71.8 year.	Intervention: Delivered via telehealth Control: Delivered in person
Kwan et al. (2020) Hong Kong	Randomised Control Trial 14 months	33 participants with cognitive frailty Mean age was 71 year	Intervention group: Conventional behavior change and mHealth (Smartphone-assisted program using Samsung Health and Whatsapp) Control group: Conventional behavior change
Jakobsson et al. (2019) Sweden	Qualitative Study	9 participants with cognitive impairment. Mean age was 74 year.	No intervention Individual semi-structured interview via telephone with Internet
Christiansen et al. (2020) Sweden	Qualitative Study 1 month	18 older adults with mild cognitive impairment or mild dementia. Median age was 78.0 year.	No intervention. Individual semi-structured interview;
Goodman-Casanova et al. (2020) Spain	Qualitative Study 1 month	100 participants with mild cognitive impairment or mild dementia.	No intervention Television-based health and social support

**Table 3:** Type of digital technology, the features and outcome measures of the selected studies.

Author/Year/ Country	Type of Digital Technology	Features	Outcomes	Remarks
Bott et al. (2018) United States	Web-based for educational material and supported by telephone, email and text messaging.	Multi-domain (Cognitive, psychosocial, physical activity, nutrition)	Primary outcome: Cognitive Secondary outcome: Depression, sleep, exercise, nutrition and stress levels.	Internet-delivered lifestyle interventions are a scalable initiative in averting or delaying of Alzheimer disease
Burton & O’Connell (2018) Canada	Video conference	Single domain (Cognitive)	Pre-post measure: neuropsychological testing and quality of life. Weakly measure: goal performance	In intervention group: Cognitive rehabilitation can be adapted to telehealth videoconferencing and showed improvement in quality of life and decreased anxiety and depression.
Kwan et al. (2020) Hong Kong	Smartphone	Multi domain (Physical activity, nutrition)	Cognitive function, frailty, walking step count, moderate-to-vigorous physical activity (MVPA)	mHealth intervention increases the walking and MVPA time of older people with cognitive frailty but larger effect was in the intervention group. mHealth is highly feasible with good recruitment, compliance and retention.
Jakobsson et al. (2019) Sweden	Mobile technology	-	Usage of technology, contact with health care, need of support, thought for future to capture participants’ experiences	Level of complexity of eHealth use are analogue use, one -way use and interactive use.
Christiansen et al. (2020) Sweden	Mobile technology	-	Meaning and purpose of mHealth, personal experience of using mHealth; Health related quality of life.	mHealth is perceived to be a supportive tool that can increase quality of life. Three categories of perceptions are require technology literacy, maintain social interaction and facilitate independent living
Goodman-Casanova et al. (2020) Spain	Television-based assistive integrated service	-	Interview; health perception-health management, sleep-rest pattern, coping-stress tolerance, physical activity and role-relationship patterns	Time of assessment, the physical and mental health and well-being of study participants was overall optimal; Television is the preferred devices to access COVID 19 information, as a recreational activity and perform memory exercise as an intellectual activity.
